# Oral pH value predicts the incidence of radiotherapy related caries in nasopharyngeal carcinoma patients

**DOI:** 10.1038/s41598-021-91600-w

**Published:** 2021-06-10

**Authors:** Zheng Li, Qiuji Wu, Xiangyu Meng, Haijun Yu, Dazhen Jiang, Gaili Chen, Xiaoyan Hu, Xinying Hua, Xiaoyong Wang, Dajiang Wang, Hongli Zhao, Yahua Zhong

**Affiliations:** 1 Department of Radiation and Medical Oncology, Zhongnan Hospital of Wuhan University, 169, Donghu Road, Wuchang District, Wuhan, 430071 Hubei Province China; 2 Hubei Key Laboratory of Tumor Biological Behaviors, Zhongnan Hospital of Wuhan University, 169, Donghu Road, Wuchang District, Wuhan, 430071 Hubei Province China; 3 Hubei Cancer Clinical Study Center Zhongnan Hospital of Wuhan University, 169, Donghu Road, Wuchang District, Wuhan, 430071 Hubei Province China; 4 Department of Urology, Zhongnan Hospital of Wuhan University, 169, Donghu Road, Wuchang District, Wuhan, 430071 Hubei Province China

**Keywords:** Cancer, Head and neck cancer

## Abstract

Radiotherapy-related caries is a complication of radiotherapy for nasopharyngeal carcinoma; however, factors influencing the occurrence, accurate prediction of onset, and protective factors of radiotherapy-related caries remain unclear. This study analyzed risk factors, disease predictors, and protective factors for radiotherapy-related caries in nasopharyngeal carcinoma. This prospective study included 138 nasopharyngeal carcinoma patients receiving radical radiotherapy at our hospital during June 2012–December 2016 and were followed up for dental caries. Patients’ clinical data on radiotherapy were collected, dynamic monitoring was performed to assess changes in oral pH values, and a questionnaire survey was administered to collect patients’ lifestyle habits. Time-dependent cox regression trees, event-free Kaplan–Meier curve, Mann–Whitely U test were used to analysis the results. The median follow-up time was 30 (12–60) months. Radiotherapy-related caries occurred in 28 cases (20.3%). Univariate analyses showed that radiotherapy-related caries was associated with patient’s age, oral saliva pH value, green tea consumption, and radiation dose to sublingual glands, but not with the radiation dose to the parotid and submandibular glands. Multivariate analysis showed that oral saliva pH value [hazard ratio (HR) = 0.390, 95% confidence interval = 0.204–0.746] was an independent prognostic factor for radiotherapy-related caries. Patients with oral saliva pH values ≤ 5.3 in the 9th month after radiotherapy represented a significantly higher risks for radiotherapy-related caries (*p* < 0.001). Green tea consumption was associated with the occurrence of radiotherapy-related caries, and oral saliva pH values could predict the occurrence of radiotherapy-related caries. Limiting radiation doses to sublingual glands can reduce the occurrence of radiotherapy-related caries.

## Introduction

Nasopharyngeal carcinoma is one of the most common malignant tumors in southern China and southeast Asia, with approximately 45,000 new cases and 24,000 deaths each year^1^. Radiotherapy is the most important treatment approach for nasopharyngeal carcinoma. A common complication of radiation to the head and neck area is radiotherapy-related caries, which severely affects patients’ quality of life. Radiotherapy-related caries tends to occur in the incisors, followed by the canines, and molars. It has a variable rate of development such that the entire crown may be damaged within weeks or years after radiotherapy^2,3^. There is an ongoing debate concerning the pathogenesis of this condition. Some scholars suggest that radiation causes direct damage to the mandible and dental structure, meanwhile it brouhgt changes in the oral microenvironment after radiotherapy by changing the function of salivary glands and the component of oral salive^4–6^. Although radiotherapy-related caries is commonly observed in clinical practice, various issues such as the factors influencing its occurrence, the accurate prediction of its onset, and protective factors remain unclear^7–9^. This study aimed to assess relevant risk factors, disease predictors, and protective factors of radiotherapy-related caries in patients with nasopharyngeal carcinoma.

## Results

### Patient characteristics

Table 1 shows exact information of all the patients. The patients included 92 males and 46 females, with an average age of 48 (12–72) years. All patients were administered intensity-modulated radiotherapy (IMRT) as planned, and 122 patients received 2–3 cycles of induction chemotherapy (docetaxel 75 mg/m^2^, D1 + cisplatin 25 mg/m^2^ D1-3, Q21d). In total, 114 patients were administered concurrent chemotherapy during radiotherapy (cisplatin 75 mg/m^2^, D1 and D22).

**Table 1:** Clinical characteristics of all nasopharyngeal cancer patients and patients developing caries.

	Number of patients	Number of patients with caries
**Sex, age**		
Male		
≤ 45 year	24	3
> 45 year	68	15
Female		
≤ 45 year	21	3
> 45 year	25	5
**T category**		
T1	19	4
T2	42	6
T3	49	11
T4	28	5
**N category**		
N0	8	3
N1	13	3
N2	106	20
N3	11	0
**Treatment**		
RT only	5	1
CT + RT	122	27
CT + RT + CT	11	0
**Radiotherapy**		
3DRT	15	9
IMRT	110	16
TOMO HD	13	1

*RT* Radiotherapy, *CT* Chemotherapy, *3DRT* 3 Dimensional conformal radiation therapy, *IMRT* Intensity modulated radiation therapy.

The median follow-up time was 30 (12–60) months. Twenty-eight patients (20.3%) suffered from radiotherapy-related caries, and the median onset time was 12(2–54) months after radiotherapy. Among them, 8 patients had dental caries in the incisor-canine region (28.58%), 6 patients had caries in the canine-molar region (21.43%), 6 patients had caries in the molar region (21.43%), and 4 patients showed full crown damage (14.29%).

### Univariate and multivariate analyses of factors influencing radiotherapy-related caries

**Figure 1:**
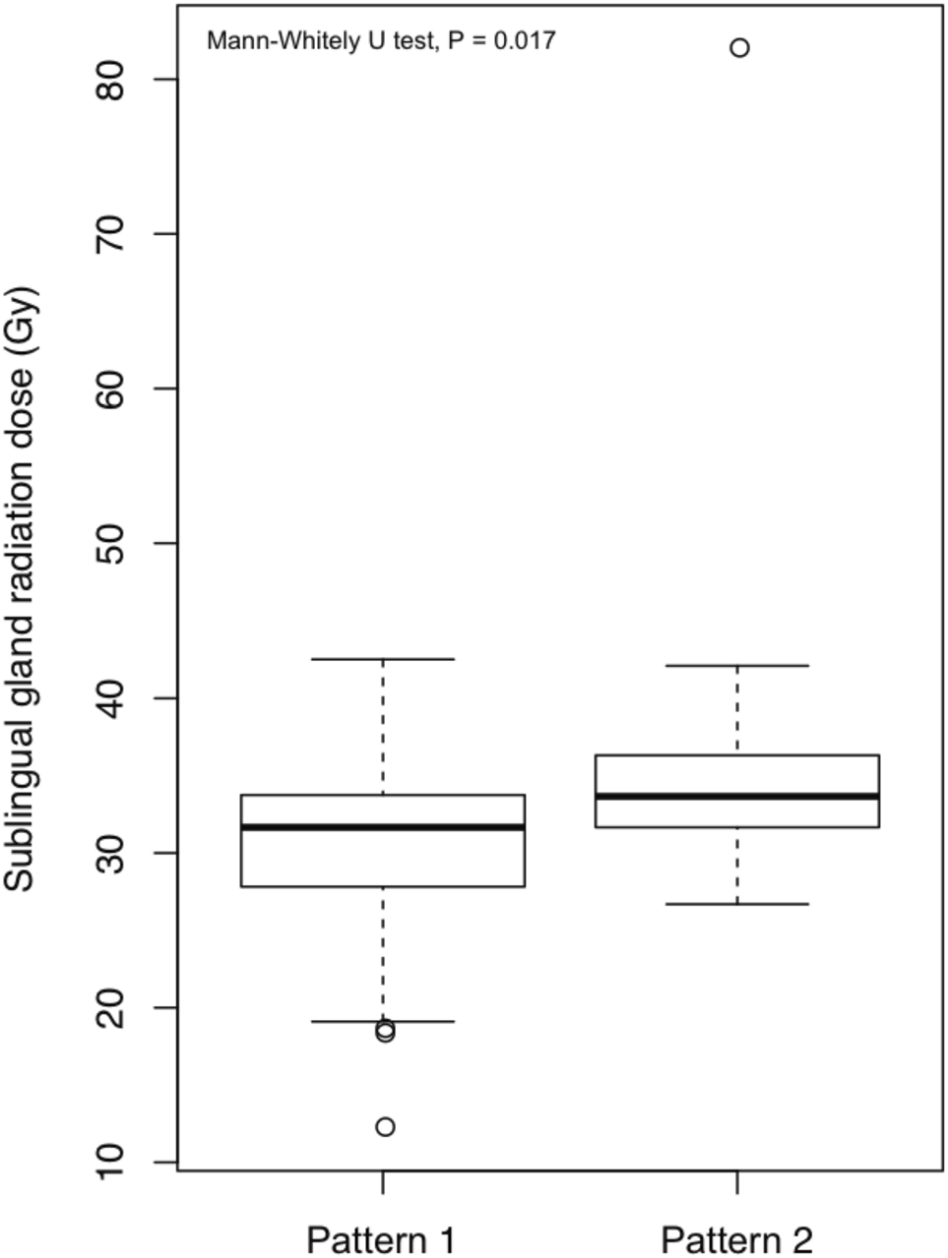
Radiation dose to sublingual glands in patients with oral pH patterns 1 and 2. Patients with oral pH value pattern 1 received a median dose of 31.66 Gy to the sublingual glands and patients with oral pH value pattern 2 received a median dose of 33.66 Gy (*p* = 0.017).

In order to investigate the factors influencing radiotherapy-related caries, this study recorded the radiation dose received by patients’ salivary glands during radiotherapy, oral saliva pH, lifestyle including dietary habits (whether they drink tea, coffee, or traditional Chinese medicine), and tooth brushing frequency based on which a time-dependent regression analysis was performed. Table 2 shows the results of univariate analyses of these factors. Oral saliva pH value [hazard ratio (HR) = 0.352], different choices of radiotherapy techniques (IMRT vs. 3DRT HR = 0.302; TOMO vs. 3DRT, HR = 0.152), dietary habits (drinking tea vs. water, HR = 2.263), and radiation dose received by the sublingual glands (HR = 1.045) had a significant effect on the occurrence of radiotherapy-related caries. In contrast, patients’ gender, disease stage, dietary habits (preference for sweet food, vegetarians vs. non-vegetarians), and radiation dose received by the bilateral parotid glands, submandibular glands, and mandible had a minimal effect on the occurrence of radiotherapy-related caries. Limiting the radiation dose received by the sublingual glands to < 32.52 Gy reduced the occurrence of radiotherapy-related caries (Fig. 1).

**Table 2:** Identification of risk factors associated with radiation-related caries by time-dependent Cox regression analysis.

	HR	95% Confidence interval	*p* value
Age (year)	1.039	1.004–1.075	0.027
Oral pH value	0.352	0.191–0.650	0.001
**Type of radiotherapy**			
IMRT versus 3DRT	0.302	0.135–0.678	0.003
TOMO versus 3DRT	0.152	0.019–1.204	0.074
**Drinking habits**			
Green tea versus water	2.263	1.075–4.764	0.032
**Dose of OAR**			
Sublingual glands	1.045	1.007–1.085	0.020
Parotid gland (le)	1.000	1.000–0.999	0.220
Parotid gland (Ri)	1.000	1.000–0.999	0.464
Mandibular	1.000	1.000–0.999	0.637
Gender	0.985	0.985–0.454	0.969
**Stage**			
T	1.143	0.783–1.666	0.489
N	0.697	0.426–1.140	0.150
Dessert	1.449	0.654–3.212	0.361
**Diet**			
Vegetarian versus others	1.994	0.878–4.529	0.099

*HR* Hazard ratio, *IMRT* Intensity-modulated radiotherapy, *3DRT* 3D conformal radiotherapy, *OAR* Organ at risk.

Further multivariate analysis for these positive factors were done (Table 3), which revealed that, oral saliva pH value was an independent prognostic factor or radiotherapy-related caries [HR = 0.390, 95% confidence interval (CI) = 0.204–0.746, *p* value = 0.04].

**Table 3:** Idenidentification of risk factors for radiation-related caries by multivariate analysis.

Factors	HR	95% Confidence interval	*p* value
Oral pH value	0.390	0.204–0.746	0.004
Age	1.018	0.979–1.059	0.367
Green tea	2.016	0.803–5.070	0.136
IMRT versus 3DRT	0.321	0.116–0.889	0.029
TOMO versus IMRT	0.328	0.032–3.335	0.346

*HR* Hazard ratio, *IMRT* Intensity-modulated radiotherapy, *3DRT* 3D conformal radiotherapy, *TOMO* Tomotherapy.

### Oral saliva pH value node

During the follow-up period of more than 4 years, we found that the oral pH values of patients may show different trends according to the different follow-up durations. Some cases showed a trend of gradual decrease, whereas others showed an initial increase, followed by a decrease. Prior to receiving radiotherapy, the majority of patients showed oral saliva pH values that fell within a range of 5.5–7.0. After radiotherapy, there was a sharp decline in patients’ oral pH values, with nearly all patients showing values below 5.0 at the first follow-up, some of whom even reached a minimum of 4.3 with time. According to the logistic regression model (Table 4), the point at which oral saliva pH value fluctuates around 5.3 is a predictor with an ideal sensitivity (71.4%) and specificity (99.1%). In order to verify this pH node value, this study divided all patients at absolute time points into two groups. The first group followed Pattern 1, wherein the patients’ oral saliva pH values were consistently > 5.3 or were initially < 5.3, but increased to > 5.3 over time. The second group followed Pattern 2, wherein the patients’ oral saliva pH values were consistently < 5.3 or were initially > 5.3 but later decreased to < 5.3. Risk comparison was performed between these two groups according to the absolute time points. All patients with Pattern 2 showed a significantly higher risk of dental caries than patients with Pattern 1 (Table 5, Supplement Fig. 1). From the 3rd to 48th month after radiotherapy, the risk value for Pattern 2 was about 15–24 times higher than that of Pattern 1. This further confirms the fact that increasing oral saliva pH value to > 5.3 could be a new measure to prevent or reduce the occurrence of radiotherapy-related caries.

**Table 4:** Identification of the optimal oral pH value for the prediction of radiation-related caries by logistic regression model.

Oral pH value	AUC	Optimal cutoff	Youden index	Sensitivity	Specificity	Positive predicted value	Negative predicted value	False positive value	False negative value
pH First	0.736 (0.643, 0.829)	5.00	0.350	0.750	0.600	0.323	0.904	0.677	0.096
pH Last	0.931 (0.838, 0.975)	5.30	0.7052	0.714	0.991	0.952	0.932	0.048	0.068

RR: Pattern 1 versus Pattern 2; Pattern 1: oral pH value always > 5.3 or from < 5.3 to > 5.3; Pattern 2: oral pH value always ≤ 5.3 or from > 5.3 to ≤ 5.3.

**Table 5:** Estimation of time-dependent risk ratios for developing radiation-induced caries throughout the follow-up period.

Follow-up length	RRC of pattern 1	Pattern 1	RRC of pattern 2	Pattern 2	RR	*p* value
3 months	8	20	20	118	14.75	< 0.001
6 months	7	19	19	117	16.71	< 0.001
9 months	7	18	18	116	16.57	< 0.001
12 months	7	16	16	103	14.71	< 0.001
15 months	4	4	4	91	22.75	< 0.001
18 months	4	3	3	87	21.75	< 0.001
24 months	3	3	3	77	25.67	< 0.001
30 months	3	2	2	53	17.67	< 0.001
36 months	3	2	2	46	15.33	< 0.001
42 months	1	1	1	24	24	< 0.001
48 months	1	1	1	22	22	< 0.001

RR: Pattern 1 versus Pattern 2; Pattern 1: oral pH value always > 5.3 or from < 5.3 to > 5.3; Pattern 2: oral pH value always ≤ 5.3 or from > 5.3 to ≤ 5.3.

### Prediction time node

The node for oral saliva pH value was obtained based on the conclusions from Table 4. By summarizing the distribution of all patients with caries, we observed that among the 28 patients with caries, more than 75% occurred within 12 months. Therefore, this study focused on analyzing the occurrence of caries within 24 months after radiotherapy, and found that the 9th month after radiotherapy during follow-up was the earliest and most optimal time period for prediction with an excellent sensitivity (77.4%) and specificity (99.1%). (Supplement Table 1, Supplement Fig. 1).

## Discussion

Radiotherapy-related caries (RRC) is a common side effect of treating nasopharyngeal carcinoma with radiotherapy. The symptoms of RRC include dentin hypersensitivity to acid and sweetness, dental erosion and tooth root fracture, leading to dysphagia, digestive difficulties and ozostomia and a significant impairment of the quality of life^2^.There is still an ongoing debate concerning the pathogenesis of radiotherapy-related caries, with some scholars suggesting that the radiation from radiotherapy can directly destroy the components, structure, and composition of the dental enamel and crown^10–12^. The radiation produced during radiotherapy can increase enamel lines, which will increase enamel damage and embrittlement; thereby, further increasing the occurrence of radiotherapy-related caries^13,14^. In this study, 8 patients had caries in the incisor-canine region (28.58%), 6 in the canine-molar region (21.43%), 6 in the molar region (21.43%), while the remaining 4 showed clear full crown damage (14.29%). The distribution of caries was not completely consistent with the region receiving high-dose radiation, which implies that direct damage from radiation was not the main cause.

Other studies have pointed out that radiotherapy-related caries may be associated with the occurrence of caries within certain mandibular regions receiving high-dose radiation^11,15,16^. The univariate analyses performed in this study found that the radiation dose received by the mandible was not correlated with the occurrence of caries (HR=1.0001; 95% CI=0.999–1.000; *p* value=0.637). Reasonable explanation may be because of the protection of dose limit of mandibular bone.

It has already been recognized that occurrence of radiotherapy-related caries is not a result of a single cause, but rather of a combination of alterations in the oral microenvironment such as oral hygiene, oropharyngeal flora, salivary functions^17^. These factors can lead to changes in salivary components, reduce organic and inorganic balanced mineral complexes, as well as increase thiocyanate content and the carboxyl groups of esters, lipids, and carbohydrates, which lead to changes in the oral microenvironment (pH, microbial flora); thereby, increasing the onset of radiotherapy-related caries^18^. This study found that changes in the oral pH played a crucial role in the pathogenesis of dental caries. The multivariate analysis indicated that oral pH value was the only factor associated with radiotherapy-related caries. We observed the changes in oral saliva pH values among patients. The oral saliva pH values before radiotherapy ranged between 5.5 and 7.0 (median = 6.5). At the first follow-up (i.e., three months after radiotherapy), the oral pH value of most patients had decreased to 4.5–5.5 (median value:5.0 ). The patients’ oral pH value increased by varying degrees over time and reached a plateau (median value: 5.5) at the 9th month of follow-up. After 12 months, the oral pH value once again increased at different rates and to different degrees. This study found that patients suffering from radiotherapy-related caries showed low levels of oral saliva pH values (4.3–5.0) for a long period after radiotherapy, and had a slow rate of recovery. Furthermore, although patients without dental caries also showed a decrease in oral saliva pH to 5.0–5.5, they recovered quickly to 5.5–6.0 and persisted in a steady state that no longer decreased. The logistic regression model (Table 4) further confirms the fact that increasing oral saliva pH value to > 5.3 could be a new measure to prevent or reduce the occurrence of radiotherapy-related caries. Risk comparison of absolute time (Table 5) showed that patients with pH lower than 5.3 was 14.71–24 times more likely than those with pH higher than 5.3 to suffer from radiotherapy-related caries. In addition, oral pH at 9 months after radiotherapy is the optimal prediction time node, with good sensitivity (81.8%) and specificity (82.7%).

Changes in the oral microenvironment are related to the radiation dose received by the parotid and submandibular glands^19–22^. Limiting the radiation dose to the parotid and submandibular glands can significantly reduce the risk and extent of xerostomia, while also protecting the oral microenvironment^20,23^. However, the exact dose limit for these glands is still unclear. The univariate analyses performed in this study did not find a correlation between the radiation dose received by the bilateral parotid glands and submandibular glands with the occurrence of caries. However, there was a correlation with the radiation dose received by the sublingual glands. The results of logistic regression analysis showed that controlling the dose received by the sublingual glands to within 32.52 Gy could reduce the incidence of radiotherapy-related caries. This fingding dose not dispute the importance of the parotid and submandibular glands in the overall microenvironment of the salivary glands. A possible reason for this finding is that patients with nasopharyngeal carcinoma must receive irradiation of the bilateral cervical lymph nodes, some even in level I reginal lymph node of neck. Hence, it is difficult to shield the bilateral parotid and submandibular glands from receiving a high radiation dose, whereas the sublingual glands can be protected by changing the beam orientation and dose limit. Therefore, among patients with nasopharyngeal carcinoma, it is essential to limit the radiation dose to the sublingual glands in order to reduce the incidence of radiotherapy-related caries.

Lifestyle habits can also affect the occurrence of radiotherapy-related caries. It was reported that living habits such as tabaco and alcohol consumption would increase the incidence of RRC^24,25^. In this study, the majority of patients quit smoking and drinking during and after RT, making it difficult to analyze the roles of these two factors. As a traditional beverage, green tea is very popular in China, especially in the Hubei Province as the traditional view that green tea has anti-oxidative and anti-tumor properties are upheld^26–30^. This study found that the pH value of normal drinking water is > 6.5, whereas that of green tea is relatively low at 5–5.5. Hence, drinking green tea may exacerbate the acidic oral environment in the long run; thereby, promoting the occurrence of dental caries.

Further potential factor which could probably affect the incidence of the radiation related caries are not mentioned because of our limited number of patients or fellow-up period.

## Conclusion

Age, xerostomia, oral pH level, type of radiotherapy, drinking habits and dose to sublingual glands are potential predictors of radiation related caries. An oral pH value lower than 5.3 is a significant risk factor. Limiting the doses to lower than 32.53 Gy to sublingual glands is suggested for the protection against RRC.

## Methods and materials

### Patients

Overall, 138 newly diagnosed patients with nasopharyngeal carcinoma who received radical radiotherapy at our hospital from June 2012 to December 2016 were enrolled in the study. The inclusion criteria were as follows: (1) pathologically confirmed malignant neoplasm of the nasopharynx (derived from epithelial cells); (2) not previously undergone radiotherapy; (4) no history of salivary gland (parotid, submandibular, or sublingual) surgery; (5) no history of oral disease or salivary gland disease; (6) no systemic disease or multiple sclerosis, xerostomia.; (7) no distant metastasis; (8) General physical condition at an acceptable level (Performance Score of 0–1 point) and an expected prognosis of survival of more than 1 year. The study was carried out in accordance with institutional guidelines and regulations and was approved by the medical ethics committee of Zhongnan Hospital of Wuhan University (No. 2017025). Written and oral informed consent was obtained from all participants or their relatives.

### Radiotherapy regimen

Images of planning computed tomography (CT)-scan with contrast were acquired with a slice thickness of 3 mm from the head to supraclavicular region. Some patients underwent position-emission tomography when necessary. The CT images were merged with contrast-enhanced magnetic resonance images (MRI). The definition and contouring of gross tumor volumes (GTV), clinical target volumes (CTV), cervical lymph node levels (II–V) and the organs at risk (OAR) including the optic nerves, chiasm, hypophysis, temporal lobes, brainstem, spinal cord, parotid glands, submandibular glands, sublingual glands, oral cavity, pharynx, larynx, thyroid gland and others were referred to the published guidelines of our departement^31^. All patients received external beam RT with 6-MV photons using an isocentric technique. The GTV of the primary tumors received 70 Gy in 31 fractions, the GTV of cervical lymph nodes 68 Gy in 31 fractions. The high risk CTV1 received 60 Gy in 31 fractions, and the CTV2 54 Gy in 31 fractions. Radiation doses were delivered one fraction per day, 5 fractions per week. Patient positioning was verified weekly by cone-beam CT for IMRT, and each time before treatment for HD tomotherapy.

### Delineation of OAR

All major salivary glands (including parotid glands, submandibular glands, sublingual glands) were delineated on multiple axial CT slices, and the sublingual glands were delineated with the help of matched MRI or PET-Scan images. Separate dose-volume histograms (DVHs) were generated for the right and left glands.

### Oral PH value measurement and RRC diagnosis

The oral pH values were assessed every 3 months during the first 2 years and every 6 months thereafter. For standardized pH measurement, patients were instructed to avoid eating and drinking for at least 60 min before saliva collection. Although the oral pH for all patients could not be measured at the same time of day, most were taken at 10 a.m. or 3 p.m,. At least 1 ml of saliva was collected and measured using the Ohaus Starter 3100C conductivity bench meter (ST3100C, Ohaus Corporation, Parsippany, NJ, USA). The salivary pH was measured by an oncologist. RRC were diagnosed independently by a radiotherapist and a dentist based on the clinical presence of caries in the participants following radiotherapy.

### Statistical analysis

We reorganized the repeatedly measured pH values into long-format. The observation period was dissected into certain intervals by follow-up schedule, and subjects’ longitudinal measurements were merged into one variable, the pH measured at each interval’s endpoint, allowing the implementation of an extended Cox regression model with pH as a time-dependent predictor. Non-time-varying factors (age, gender,) were passed to univariate Cox regression. We then conducted multivariate analysis taking pH the predictor of interest and adjusted for covariates of significance in univariate analyses. We identified the optimal cut-off of pH by Cox regression tree, dichotomized the subjects into high and low pH groups, and tested its predictive power. We further explored the dynamics in its predictive power with logistic regression by different follow-up lengths, and calculated corresponding odds ratios (OR). Then, we examined the patterns of time-varying pH group status over the observation period of each subject, summarized them into a simple dichotomous variable, analyzed it in Cox models, and calculated risk ratios (RRs) for caries between the two patterns by lengths of follow-up and event-free survival by Kaplan–Meier curve. ROC curves from Logistic regressions were used to determine the optimal cutoff value by maximizing Youden Index. Time-to-Cary-Occurrence analysis were also performed using the Kaplan–Meier estimator and the log-rank test.Hazard ratios (HRs), their 95% confidence intervals (95% CIs) and concordance indices were calculated for Cox models. A *p* < 0.05 indicated statistical significance, performed using the R 3.4.3.

## Supplementary Information

Supplementary Information.
