# Supplementary material for: Oral pH value predicts the incidence of radiotherapy related caries in nasopharyngeal carcinoma patients
Source: Sci Rep. 2021 Jun 10;11:12283. doi: 10.1038/s41598-021-91600-w (PMC8192759; doi:10.1038/s41598-021-91600-w)
Supplement: Supplementary file 1 — Supplementary Information. [file 41598_2021_91600_MOESM1_ESM.docx]

**Oral pH value predicts the incidence of radiotherapy related caries in nasopharyngeal carcinoma patients**

Zheng LI,PhD^1-3^**^†^**; Qiuji WU, PhD^1-3^**^†^**; Xiangyu MENG, PhD^4^; Haijun YU, PhD^1-3^; Dazhen JIANG, Master^1-3^; Gaili CHEN, PhD^1-3^; Xiaoyan HU, PhD^1-3^;Xinying HUA, Master^1-3^; Xiaoyong WANG, Master^1-3^; Dajiang WANG, Master ^1-3^; Hongli ZHAO, Master^1-3^; Yahua ZHONG, PhD^1-3*^

^1^ Department of Radiation and Medical Oncology, Zhongnan Hospital of Wuhan University. 169, Donghu Road, Wuchang District, Wuhan, Hubei Province, 430071, China.

^2^ Hubei Key Laboratory of Tumor Biological Behaviors Zhongnan Hospital of Wuhan University. 169, Donghu Road, Wuchang District, Wuhan, Hubei Province, 430071, China.

^3^ Hubei Cancer Clinical Study Center Zhongnan Hospital of Wuhan University. 169, Donghu Road, Wuchang District, Wuhan, Hubei Province, 430071, China.

^4^ Department of Urology, Zhongnan Hospital of Wuhan University. 169, Donghu Road, Wuchang District, Wuhan, Hubei Province, 430071, China.

**^†^** Co-first author

^*^ Corresponding author: Yahua ZHONG

E-mail: doctorzyh73@163.com

Tel: +86-13207100729

Fax: +86-027-6781-3162

**Supplementary files**


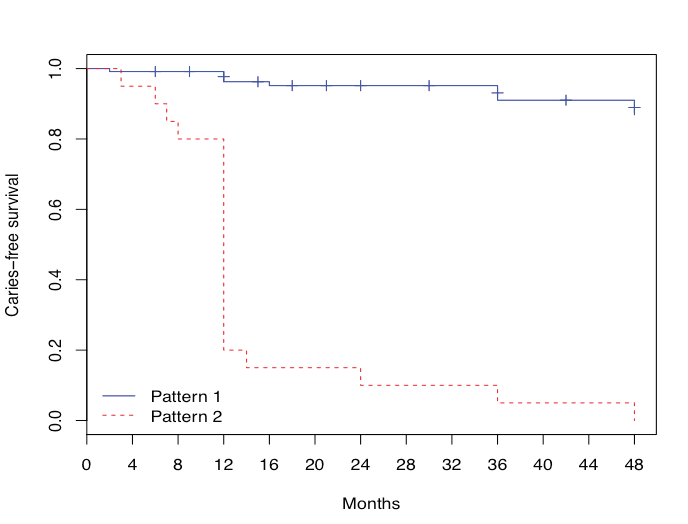


**Supplement Figure 1 Event-free survival of patients with oral pH patterns 1 and 2.** Pattern 1: oral pH value always >5.3 or from <5.3 to >5.3; Pattern 2: oral pH value always <=5.3 or from >5.3 to <= 5.3. p<0.05.


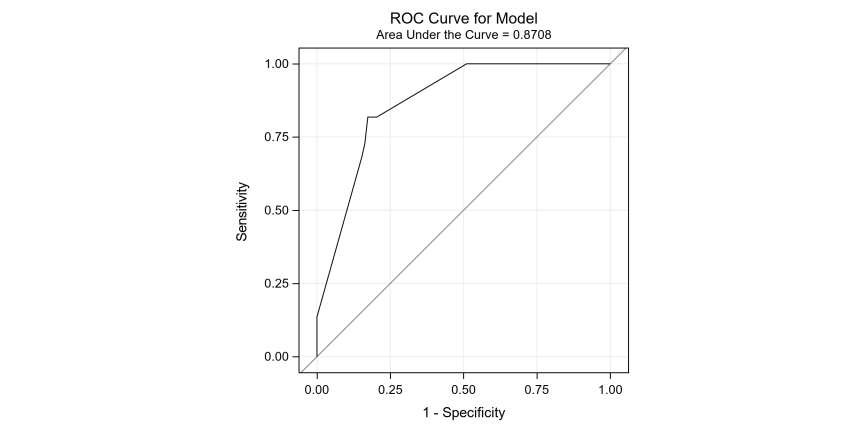


**Supplement Figure 2 Receiver Operating Curve (ROC) of pH values at 9^th^ month was obtained when predicting the incidence of radiotherapy-related caries.** Area under the curve was 0.8708.

Supplement Table 1. Performances of oral pH values at specific timepoints using the Logistic regression mode.

| Paramter | AUC | Optimal Cutoff | Youden  Index | Sensitivity | Specificity | Positive Predicted | Negative Predicted Value | False  Positive  Value | False  Negative  Value |
| --- | --- | --- | --- | --- | --- | --- | --- | --- | --- |
| 3 months | 0.736(0.643,0.829) | 5.00 | 0.350 | 0.750 | 0.600 | 0.323 | 0.904 | 0.677 | 0.096 |
| 6 months | 0.7696(0.704,0.890) | 5.30 | 0.474 | 0.738 | 0.743 | 0.404 | 0.921 | 0.596 | 0.080 |
| 9 months | 0.871(0.807,0.935) | 5.30 | 0.645 | 0.818 | 0.827 | 0.514 | 0.529 | 0.486 | 0.047 |
| 12 months | 0.832(0.716,0.948) | 5.30 | 0.615 | 0.833 | 0.781 | 0.588 | 0.926 | 0.412 | 0.074 |
| 15 months | 0.896(0.744,1.000) | 5.20 | 0.683 | 0.750 | 0.933 | 0.600 | 0.966 | 0.400 | 0.035 |
| 18 months | 0.982(0.941,1.000) | 5.40 | 0.927 | 1.000 | 0.927 | 0.571 | 1.000 | 0.429 | 0.000 |
| 21 months | 1.000(1.000,1.000) | 5.30 | 1.000 | 1.000 | 1.000 | 1.000 | 1.000 | 0.000 | 0.000 |
| 24 months | 0.927(0.810,1.000) | 5.50 | 1.000 | 1.000 | 0.683 | 0.188 | 1.000 | 0.813 | 0.000 |
